# ObScene database: Semantic congruency norms for 898 pairs of object-scene pictures

**DOI:** 10.3758/s13428-023-02181-7

**Published:** 2023-07-24

**Authors:** Miguel Ângelo Andrade, Margarida Cipriano, Ana Raposo

**Affiliations:** Research Center for Psychological Science, Faculdade de Psicologia, Universidade de Lisboa, Alameda da Universidade, 1649-013 Lisboa, Portugal

**Keywords:** Norms, Semantic relationship, Congruency, Object, Scene, Real-world picture

## Abstract

Research on the interaction between object and scene processing has a long history in the fields of perception and visual memory. Most databases have established norms for pictures where the object is embedded in the scene. In this study, we provide a diverse and controlled stimulus set comprising real-world pictures of 375 objects (e.g., suitcase), 245 scenes (e.g., airport), and 898 object–scene pairs (e.g., suitcase–airport), with object and scene presented separately. Our goal was twofold. First, to create a database of object and scene pictures, normed for the same variables to have comparable measures for both types of pictures. Second, to acquire normative data for the semantic relationships between objects and scenes presented separately, which offers more flexibility in the use of the pictures and allows disentangling the processing of the object and its context (the scene). Along three experiments, participants evaluated each object or scene picture on name agreement, familiarity, and visual complexity, and rated object–scene pairs on semantic congruency. A total of 125 septuplets of one scene and six objects (three congruent, three incongruent), and 120 triplets of one object and two scenes (in congruent and incongruent pairings) were built. In future studies, these objects and scenes can be used separately or combined, while controlling for their key features. Additionally, as object–scene pairs received semantic congruency ratings along the entire scale, researchers may select among a wide range of congruency values. ObScene is a comprehensive and ecologically valid database, useful for psychology and neuroscience studies of visual object and scene processing.

## Introduction

In our daily lives, everything we look at constitutes a scene, a scenario, a picture, a visual environment, which integrates different features and items. Thus, it is no wonder that visual stimuli are so important in the study of human cognition and behavior. The use of pictures as experimental stimuli has a long history in social and cognitive psychology, as well as in cognitive neuroscience. Compared to words, the visual processing of pictures starts earlier in life (Whitehouse et al., 2006), and their semantic processing is not dependent on linguistic or conceptual development (Clark, 1995; Whitehouse et al., 2006). Pictures are more realistic and present a richer variability in physical aspects, closer to our natural environmental stimuli (Kovalenko et al., 2012; Öhlschläger & Võ, 2017), and are better remembered than words (Dewhurst & Conway, 1994; Snodgrass & Vanderwart, 1980).

Isolated objects have been the most common items represented in pictorial stimuli, with several databases available in the literature, from the pivotal database by Snodgrass and Vanderwart (1980) containing line drawings of objects, to more realistic databases of photographs of objects (e.g., Brodeur et al., 2014; Moreno-Martínez & Montoro, 2012; Souza et al., 2021). In real environments, we see and interact with objects embedded in a context or background, with objects and scenes being processed not independently, but rather interactively (Davenport & Potter, 2004). Consequently, databases of objects within background scenes have also been developed, promoting more ecologically valid research (Bar, 2004; Bates et al., 2003; Duñabeitia et al., 2018; Hebart et al., 2019; Krautz & Keuleers, 2022; Szekely et al., 2004; Võ, 2021).

An important factor underlying the joint processing of objects and scenes concerns their association, which is established by our acquired knowledge along time and through previous experiences. A consistent association between an object and a scene (e.g., a piano and a concert hall) creates a statistical regularity in the surrounding environment, and makes us expect to find certain objects within certain scenes based on probability (Bar, 2004, 2021; Shir et al., 2021). The predictable property of this relationship is based on preexisting semantic knowledge and has been called *semantic congruency* (e.g., Kovalenko et al., 2012; Shir et al., 2021) or *semantic consistency* (e.g., Davenport & Potter, 2004; Öhlschläger & Võ, 2017). The literature shows that when objects are found within congruent contexts they are more accurately and rapidly recognized (e.g., Biederman et al., 1982; Davenport & Potter, 2004; Oliva & Torralba, 2007; Palmer, 1975). Conversely, semantic incongruencies occur when objects do not fit in a particular scene (e.g., a piano and a parking lot). Such semantic violations or violations of semantic expectations (Kovalenko et al., 2012; Öhlschläger & Võ, 2017; Shir et al., 2021) have been described as a violation of part of the *scene grammar*, as the object disrupts the scene global meaning (see also Biederman et al., 1982; Võ & Wolfe, 2013). This scene grammar also contemplates “syntactic” properties of a picture, related to the expected physical position of the objects in the scene (e.g., a piano is expected to be on stage, not in the audience), although these properties are out of the scope of this study (for a database focused on these characteristics, see Mohr et al., 2016).

Object–scene semantic congruency has been studied using different types of pictures, from line drawings (Biederman et al., 1982; De Graef et al., 1990; Henderson et al., 1999; Hollingworth & Henderson, 2000; Palmer, 1975), to photographs of real-world scenes (Coco et al., 2020; Proverbio & Riva, 2009; Underwood et al., 2008; Underwood & Foulsham, 2006), or digitally generated images (Davenport & Potter, 2004; Demiral et al., 2012; Draschkow et al., 2018; Mudrik et al., 2010; Underwood et al., 2007). Despite extensive research using a combination of object and scene pictures, normative data for scenes are relatively scarce, particularly scene databases validated for the same variables as most object databases. Thus, the first goal of the present study is to create a database of object and scene pictures, normed for the same variables in order to have comparable measures for both types of pictures. The second goal is to acquire normative data for the semantic relationships between the objects and the scenes. As discussed in more detail below, a number of studies have provided norms for the semantic relationship between objects and scenes, with the object embedded in the scene. In the present database, the objects and the scenes are presented separately. This offers greater flexibility in the use of pictures that can be used alone or combined in several ways. It also allows disentangling the processing of the object and its context (the scene), which is useful for researchers working in cognitive domains such as memory and language.

The construction and validation of this type of database is extremely valuable for experimental research. There has been a growing concern on how good and well controlled the visual stimuli employed across studies are, with several normative studies being published in the past few decades (e.g., Souza et al., 2020). Creating a stimulus set is highly demanding and requires thorough examination, particularly when using real-world pictures (Shir et al., 2021). It requires resources and knowledge on one hand, but saves time and effort for future investigations on the other hand. First, there is the need to search for and select a considerable number of images following strict criteria; then, in the case of relationships between images, it is necessary to combine the pictures (e.g., in pairs); and finally, submit the stimuli for judgment by participants in order to confirm the experimenters’ choices, and to classify and validate relevant image properties and variables of interest (Shir et al., 2021).

### Existing object–scene databases

Recently, Shir et al. (2021) have built the *ObjAct* stimulus set, comprising 120 photographs of scenes, in which a congruent and an incongruent object were digitally inserted so the object is embedded in the scene. Each scene includes two representations for each type of semantic congruency (congruent and incongruent). Participants’ ratings confirmed that congruent images were considered significantly less “weird” and more likely to appear in the real world (Shir et al., 2021). However, all stimuli in this database represent actions being performed with those objects by humans, who integrate the scene context (for a previous study using the same type of material, see Mudrik et al., 2010). Unlike other action databases, there is a particular focus on the objects’ congruency and not on the plausibility of the whole scene being represented (e.g., having dinner in the water; Riva et al., 2020). Nonetheless, restricting object–scene relations to the performance of actions may involve cognitive processes associated with movement and motor cognition, along with the recruitment of motor areas (for reviews on motor cognition and the neural representation of actions, see Jeannerod, 2001, 2006). Moreover, by including both human faces and letters/words in some of the images, the stimuli may induce confounds and/or interfere with the object–scene congruency manipulation, as it is well-known that faces and words have dedicated cognitive processes (e.g., Diaz & McCarthy, 2007; Farah et al., 1998; James & Gauthier, 2006; Posamentier & Abdi, 2003) and frequently direct the attention of the observer (e.g., Sreenivasan et al., 2009; Valenza et al., 2014; Wu et al., 2014).

In another study based on the previously mentioned scene grammar perspective, Öhlschläger and Võ (2017) built the *SCEGRAM* stimulus set, consisting of 62 scenes, with each scene being associated with six conditions. In half of the conditions, the scene was photographed with a semantically congruent object embedded, while in the other half a semantically incongruent object was embedded. Additionally, the authors included a syntactic manipulation of the object’s position in the scene, which could be expected, unexpected, or physically impossible. Participants’ ratings confirmed a significant difference in consistency between the semantically congruent and incongruent conditions. This database also includes the photographs of the same 62 scenes without the critical objects, as well as of the 62 objects alone on a white background. However, the semantic relationship between the objects and scenes, when displayed separately, was not tested. Also, even though the stimuli do not include actions or human faces, some of the critical objects contain verbal information, such as letters or words. Besides, it is unclear if the critical objects or the other objects in the scenes are repeated across different scene images, which could represent a caveat as repetition impacts object recognition.

Of note, an important advantage of the aforementioned databases is the use of toolboxes to evaluate low-level image features (such as luminance, contrast, color). Yet, they lack an evaluation of three key variables within picture databases, i.e., name agreement, familiarity, and visual complexity, which, in a recent review, have been identified as some of the most commonly validated variables in object picture databases (Souza et al., 2020). The same is the case for some scene databases available (e.g., Greene, 2013; Jiang et al., 2022; Konkle et al., 2010; Saraee et al., 2018; Xiao et al., 2010). In a survey with eight subjective judgments about images, Shir et al. (2021) inspected the visual complexity of the pictures (i.e., “How visually complicated is the image?”), though it is possible that visual complexity (i.e., the amount of visual detail present within the image) may be confounded with the complexity of the action being portrayed (which may depend on sensorimotor integration and social understanding skills).

To the best of our knowledge, no database to date has presented normative data for semantic congruency ratings between objects and scenes presented as separate pictures, i.e., without the object embedded in the scene. Furthermore, although there are numerous databases of object pictures, there is a lack of normative studies of scene pictures alone, and particularly scene databases validated for the same variables as most object databases. Moreover, several of the scene pictures available include actions, human faces, or letters which may induce attentional bias towards these elements, constituting potential confounds. We took these methodological limitations into consideration and attempted to overcome them in the current study.

### Implications for and application to human cognition

For decades, object and scene pictures have been used to study a wide range of mnemonic processes. Often, these studies produce stimuli anew, rather than taking advantage of existing databases. This is problematic, as materials vary considerably across studies and on many occasions the stimuli are not adequately normed, increasing the chances of confounding effects.

For research fields such as perception, visual search, or object recognition, it is certainly useful to have scene databases available that include the target object as part of the background image, i.e., embedded in the scene (as in the databases cited above). However, for memory research this might create confounds between perception and memory processing. This concern becomes evident, for example, in the vastly investigated effect of context on item memory retrieval (e.g., Boyce & Pollatsek, 1992; Hayes et al., 2007; Hollingworth, 2006; Mandler & Johnson, 1976; Ngo & Lloyd, 2018; van Kesteren et al., 2013). Since the main goal is to study memory (and not perception or object recognition), it is important to ensure that participants do not waste encoding time in visual searching, looking for the target object in a naturally complex scenario. Besides, it is recommended that, across trials, participants spend the same amount of time encoding the items. Importantly, the literature has consistently shown that object recognition is impaired when objects are embedded in a coherent or congruent scene (Bar, 2004; Davenport & Potter, 2004; Murphy & Wisniewski, 1989; Spaak et al., 2020), which is problematic when investigating item or context memory for congruent versus incongruent object–scene pairs.

These concerns might explain why an object–scene configuration with the target object embedded in a background scene is rarely used in memory research. Instead, most frequently, researchers have used two separate images: one for the target object and another one for the scene. The preferred object–scene configuration has been the image of an object in a white background superimposed in the context scene (e.g., Fandakova et al., 2017; McAndrews et al., 2016; Sastre III et al., 2016; Selmeczy et al., 2019; Wang et al., 2018). Sometimes, an object image without background is digitally inserted in the scene, but in those cases the target object is very salient (e.g., Hayes et al., 2007; Ngo & Lloyd, 2018). Alternatively, the object and scene pair are displayed side by side (e.g., Brod & Shing, 2019; van Kesteren et al., 2013), which allows the rapid identification of the items that participants must attend to during encoding and subsequent retrieval.

Another cognitive domain that can benefit from validated stimuli of objects and scenes presented separately is language. Pictures of objects are frequently used in the visual world paradigm and in word–picture verification tasks in psycholinguistic studies. As pointed out by Henderson and Ferreira (2004), scene perception is also crucial for language research to investigate how the visual world is perceived and how it bears on language processing. In fact, recent evidence has revealed that, in a sentence–picture verification task, participants’ recognition of the target picture depends on the semantic similarity between the scene evoked by the sentence and the scene presented in the picture (Horchak & Garrido, 2022). Thus, the current database should be of interest for language comprehension studies.

### Present study

The current study presents a systematic validation of real-world pictures of objects and scenes, as well as their semantic congruency. It adds to the existing datasets of naturalistic object and scene images, making two main contributions: (1) creating a database of object and scene pictures, normed for the same variables, thus offering comparable measures for both types of pictures; (2) providing normative data for the semantic congruency between the objects and the scenes that are presented separately (rather than embedded). In this way, the objects and the scenes can be used more flexibly, in isolation or combined. The images can also be employed in studies that aim to disentangle the processing of the object and its context (the scene), while manipulating their relationship, a key feature in memory and language research.

Semantic congruency ratings between real-world pictures of objects and scenes were acquired for a total of 898 object–scene pairs, conducted with a Portuguese sample of young, healthy adults. Two precautions were taken into account: the images do not include any letters, words, or human faces, and the objects that constitute the stimuli do not appear in any of the scene images. In order to create a comprehensive and ecologically valid object–scene database, it is important to validate the degree of semantic congruency of each object–scene pair, and to characterize each one of the pictures that integrate the stimulus set. Due to the inherent complexity and variability of visual stimuli, especially of real-world pictures like photographs, different picture properties should be assessed. As mentioned before, three important variables assessed in picture databases are name agreement, familiarity, and visual complexity (e.g., Brodeur et al., 2014; Cycowicz et al., 1997; Snodgrass & Vanderwart, 1980; for a systematic review on object databases, see Souza et al., 2020). *Name agreement* represents the most chosen (i.e., modal) name that the sample of participants attributes to the concept being portrayed in the picture or photograph (e.g., Brodeur et al., 2010; Snodgrass & Vanderwart, 1980; Souza et al., 2021); in our case, the name of an object or of a scene. With this measure, we obtain both the modal name and the respective proportion of choice among participants. *Familiarity* represents the level of interaction a person has had with the represented concept. This interaction can be either through physical or visual contact, or by thinking about the item (e.g., Brodeur et al., 2010; Snodgrass & Vanderwart, 1980; Souza et al., 2021). *Visual complexity*, unlike the previous measures, is a variable directly associated with the image and not with the concept being represented. It reflects the level of detail and quantity of surface features displayed in the image (e.g., Brodeur et al., 2010; Snodgrass & Vanderwart, 1980; Souza et al., 2021). According to Souza et al. (2021), these features may include color, shape, brightness, luminosity, contrast, size, or line complexity. These three variables often correlate with each other. Notably, familiarity tends to correlate positively with name agreement and negatively with visual complexity (Brodeur et al., 2014; Moreno-Martínez & Montoro, 2012; Snodgrass & Vanderwart, 1980; Souza et al., 2021).

In Experiment 1, we collected normative data for 620 color pictures, including photographs of 375 common objects and 245 common scenes on the three variables described above. Furthermore, we presented two different exemplars of a subset (*N* = 120) of the scenes (e.g., two kitchen scenes). This represents an additional asset of this database, as in future studies two instances of the same semantic concept may be presented with the purpose of disentangling between the target scene and a related lure (e.g., in a two-alternative forced choice paradigm as in Konkle et al., 2010). In Experiment 2, each one of a total of 125 scenes was paired with three semantically congruent objects and another three semantically incongruent objects, composing picture septuplets. Participants rated the semantic congruency of each of these 750 relationships. Finally, in Experiment 3, each of 120 objects was paired with two different scenes, constituting picture triplets totaling 240 semantic relationships, where either both scenes were semantically congruent with the object, both scenes were incongruent with the object, or one scene was congruent and one incongruent with the object. As a subset of the object–scene pairs had already been tested in Experiment 2, in Experiment 3, participants only rated the congruency of the remaining 148 pairs. The organization of the material in septuplets and triplets in the last two experiments allows, in future studies, the choice of different semantic relationships with multiple levels of congruency for the same scene or object. Besides, considering the variables evaluated in Experiment 1, it is possible to manipulate or control the chosen semantic relationships in accordance with the pictures’ individual parameters.

## Experiment 1

### Method

#### Participants

A group of 191 young adults participated in this experiment (164 female, *M*_age_ = 19.5 years, age range = 18–32 years). They were all university students in Portugal. All participants provided oral informed consent, had European Portuguese as their native language, and had normal or corrected-to-normal vision. The experimental procedures were approved by the local ethics committee.

#### Stimuli

**Fig. 1:**
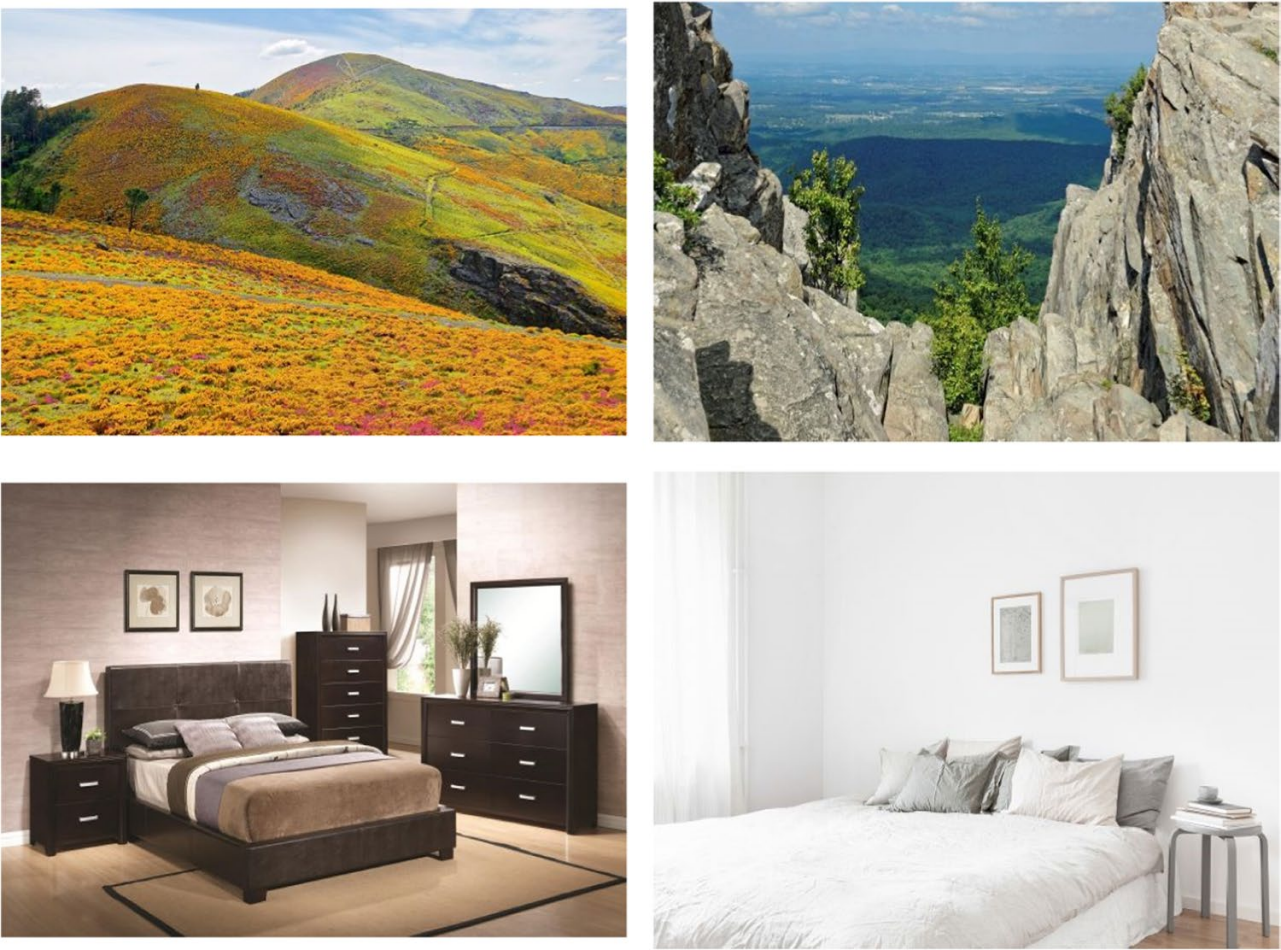
Illustration of the two instances of the “Mountain” and the “Bedroom” scenes

A dataset of 620 color photographs was built by selecting images from the Google Images dataset. To ensure that no copyrights were violated, the filter “labeled for reuse with modifications” was selected. Most pictures did not contain any human faces, words, or isolated letters at the time of selection. When these elements were present (in a reduced number of images), they were removed using Adobe Photoshop software. The picture set included 375 common objects (all non-living items, except for a cactus picture) and 245 common scenes (140 indoor and 105 outdoor). The scenes depicted places (i.e., real-world environments) with no actions portrayed. Importantly, none of the 375 objects appeared in the scene pictures. Additionally, the 245 scenes represented 125 distinct places: 120 places with two different scene exemplars each (e.g., two kitchens) and five places with only one instance. The two exemplars of the same scene were chosen to be as visually different as possible from each other (see Fig. 1 for examples). All images can be found in the following Open Science Framework project: https://osf.io/4pqsu/?view_only=9478429999754bd1afc3823c4876de18.

Regarding the image size, we adopted a different criterion for objects and scenes, since the two types of images represent very distinct size scales. Object images were resized individually in order to reach the approximate proportion between objects in real life. Size constancy is a relevant part of our semantic knowledge about objects and an important factor in object perception (Kristensen et al., 2021). This rationale is also in accordance with the “familiar size” rule of the object’s organization in the real world (Biederman et al., 1982) and with the finding that objects have a consistent size at which they are drawn, imagined, and viewed, and critically this size is proportional to the real size of the object (Konkle & Oliva, 2011). Consequently, the images’ width varied between 64 and 450 pixels, and their height between 65 and 400 pixels. All objects were presented on a white background. Scene images were resized to fixed 600 pixels in width, allowing the height to vary according to each image proportion (between 337 and 524 pixels).

#### Procedure

Participants were tested in small groups at the university laboratories. One third of the participants saw the images on the computer screen and answered manually using paper and pencil, and two thirds responded within an experiment built in E-Prime software (Psychology Software Tools, Sharpsburg, PA, USA) using the keyboard of the computer. At the beginning of the experiment, after giving informed consent, participants provided sociodemographic information (i.e., age, gender, and native language).

Participants had to perform a triple-trial task on each image presented in the following order: (1) name agreement task, (2) familiarity rating task, and (3) visual complexity rating task. In the name agreement task, participants were asked to write the name of the object or scene that they identified in the picture. For the familiarity assessment, they were instructed to rate on a five-point Likert scale (1 = very unfamiliar, 5 = very familiar) how familiar the concept depicted in each image was, considering the degree of usual physical or visual contact, and usual thought or knowledge about that particular object/place. In the case of visual complexity, participants were required to rate on a five-point Likert scale (1 = very simple, 5 = very complex) the amount of visual detail and the intricacy of lines, patterns, or features of the image, regardless of the object or place being portrayed. Given their distinct nature, objects and scenes were evaluated either by different participants or in different experimental blocks. The experimenter gave oral examples (with objects/scenes not included in the test) to each of the variables and participants responded at their own pace to the questions. The number of participants per picture varied between 17 and 34 for objects (*M*_N_ = 22.1, *SD* = 5.4) and 25 and 29 for scenes (*M*_N_ = 27.2, *SD* = 1.1).

### Results and discussion

Data preprocessing and analysis was performed by item type (object or scene) and, for each one of the 620 pictures, a qualitative dimension was obtained (i.e., the most common or modal name), as well as three quantitative parameters (i.e., percentage of modal name agreement, familiarity mean rating, and visual complexity mean rating).

**Fig. 2:**
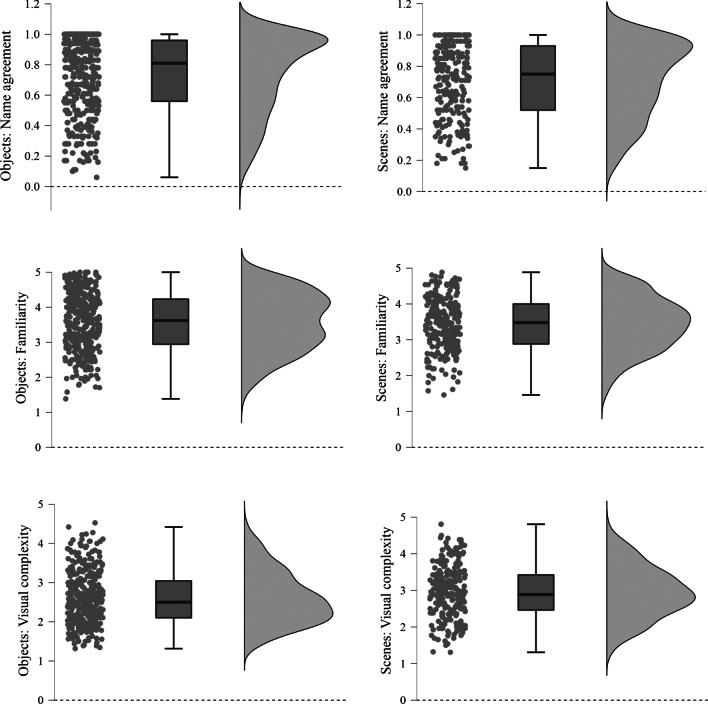
Distributions of the quantitative parameters of the object and scene pictures. *Note*. Top: Distributions of name agreement proportions. Middle: Distributions of mean familiarity. Bottom: Distributions of mean visual complexity. Left: Distributions of objects. Right: Distributions of scenes. Each graph includes individual dots (corresponding to individual images), boxplots, and raincloud plots (Allen et al., 2019) of each distribution. For a detailed data overview, see Tables S6 and S7 on the online supplemental materials (https://osf.io/4pqsu/?view_only=9478429999754bd1afc3823c4876de18)

For the qualitative dimension, the preprocessing procedure started with an examination of basic variants of the same name (e.g., plural, gender, order of composite names) and eventual spelling mistakes (following Brodeur et al., 2014; Souza et al., 2021). Even though there were significant differences in the ratings between the two response versions (i.e., higher familiarity ratings in the paper–pencil version and higher visual complexity ratings in the computer-based version), the results revealed strong correlations between the two in all the dimensions tested, for both objects and scenes (all *r*s > .86, *p*s < .001). We therefore opted for presenting the conjoint results (collapsing across the paper–pencil and computer-based versions). Descriptive statistics for the three quantitative parameters for both objects and scenes are depicted in Fig. 2. Detailed information and descriptive statistics for each item can be found in Table S1 (objects) and Table S2 (scenes) as online supplemental materials (https://osf.io/4pqsu/?view_only=9478429999754bd1afc3823c4876de18).

Participants showed high modal name agreement for both objects and scenes (*M*s > 70%). Overall, object familiarity was significantly above the scale midpoint, whereas visual complexity was below it (scale midpoint = 3; *p*s < .001), in line with earlier work (Souza et al., 2021). In the case of scenes, familiarity was also above the scale midpoint (*p* < .001), but visual complexity was not significantly different from it (*p* > .05). All parameters followed a normal distribution with suitable values for both skewness and kurtosis (i.e., between ±2; Gravetter & Wallnau, 2014).

In order to compare the dimensions across the two types of items, we ran independent-samples *t*-tests between the object and scene parameters. We found no significant difference regarding name agreement (*p* > .05), but objects were more familiar (*p* = .044) and visually less complex (*p* < .001) than scenes overall. We next compared objects and indoor and outdoor scenes. Results were considered significant if exceeding an alpha threshold of .017 (Bonferroni corrected for multiple comparisons). As in the previous analysis, objects were considered visually less complex than both indoor (*p* < .001) and outdoor scenes (*p* = .007). However, objects did not show a significantly different familiarity level from indoor (*p* = .235) or outdoor scenes (*p* = .043). The direct comparison between indoor and outdoor scenes revealed no significant differences in the proportion of name agreement or familiarity ratings (*p*s > .05). Nevertheless, indoor scenes (*M* = 3.04, *SD* = .69) revealed higher visual complexity ratings than outdoor ones (*M* = 2.80, *SD* = .69; *p* = .008). This is presumably due to the higher number of objects within indoor scenes (Greene, 2013), as well as the fact that indoor scenes reflect human-made environments, which in turn contain more details and are more complex than outdoor/natural environments.

We subsequently inspected potential correlations between the three quantitative parameters using Pearson correlation analyses, for objects (Table 1) and scenes (Table 2) separately.

**Table 1:** Correlation matrix between the quantitative parameters for the objects

	Name agreement	Familiarity
Familiarity	.413 ***	
Visual complexity	−.044	−.275 ***

*** *p* < .001 (two-tailed)

**Table 2:** Correlation matrix between the quantitative parameters for the scenes

	Name agreement	Familiarity
Familiarity	.350 *	
Visual complexity	−.108	−.079

* *p* < .05 (two-tailed)

As expected, for objects, familiarity correlated positively with name agreement (*r*(375) = .41, *p* < .001) and negatively with visual complexity (*r*(375) = −.28, *p* < .001). Hence, less complex items tend to be more familiar, and the higher an item’s familiarity the higher is the consensus on the attributed name. These findings are in agreement with most studies involving object pictures (e.g., Brodeur et al., 2014; Moreno-Martínez & Montoro, 2012; Snodgrass & Vanderwart, 1980; Souza et al., 2021). In the case of the scenes, only a significant and positive correlation emerged between familiarity and name agreement (*r*(73) = .27, *p* = .020). Thus, as previously observed with objects, increasing familiarity of the scene was associated with increasing naming agreement.

## Experiment 2

### Method

#### Participants

A group of 88 young adults, who did not participate in Experiment 1, participated in this experiment (71 female, *M*_age_ = 19.9 years, age range = 18–32 years). They were all university students in Portugal. They all provided oral informed consent, had European Portuguese as their native language, and had normal or corrected-to normal-vision. The experimental procedures were approved by the ethics committee of the same university.

#### Stimuli

**Fig. 3:**
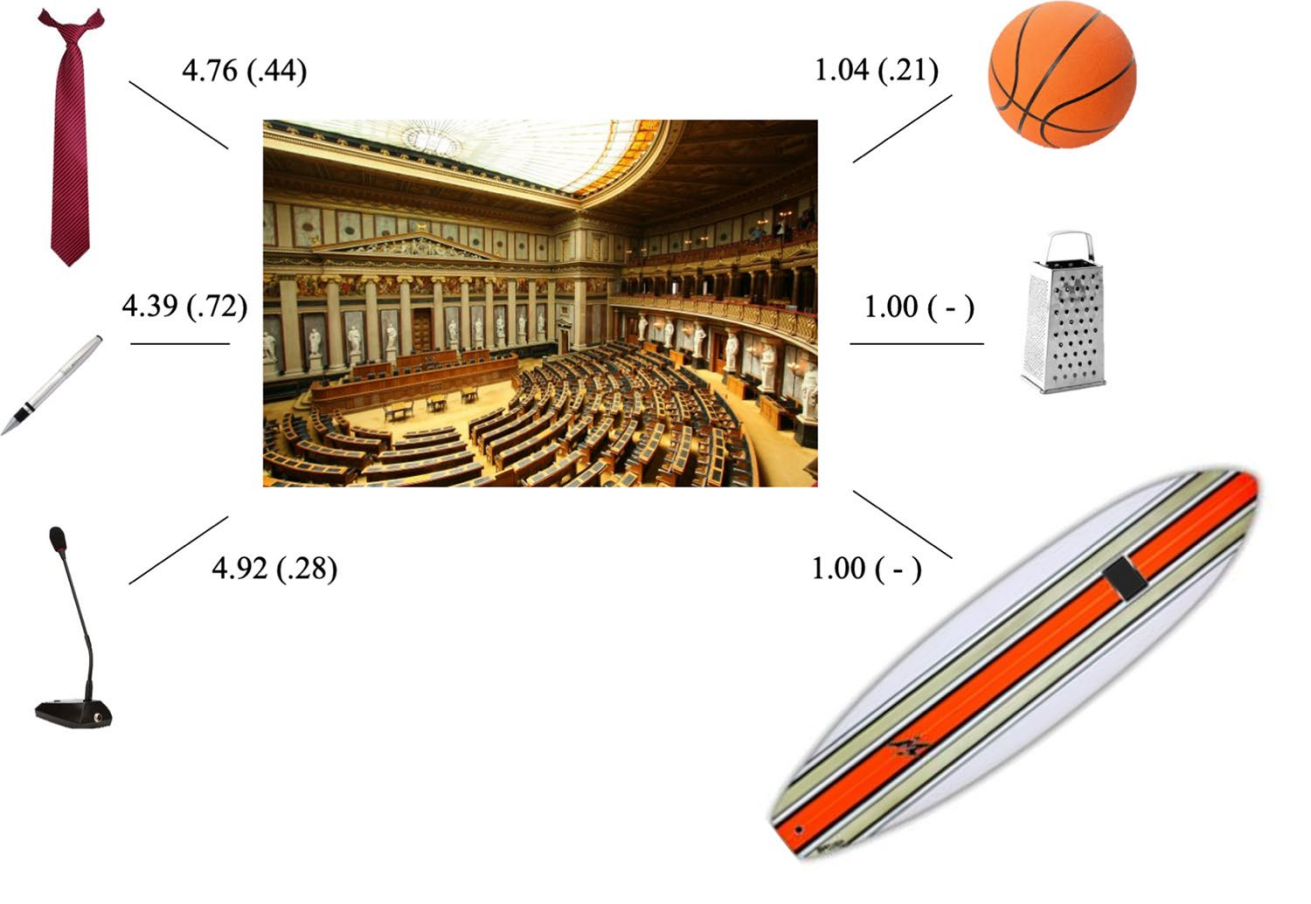
Illustration of one of the septuplets consisting of one scene and six objects. *Note.* Three objects (on the left; i.e., a tie, a pen, and a desk microphone) are congruent with the scene (i.e., a parliament) and three objects (on the right; i.e., a basketball, a grater, and a surfboard) are incongruent with the same scene. Each value represents the mean congruency rating (and standard deviation) for that particular object–scene pair. A null standard deviation means that the rating was constant across participants

We used 500 pictures from Experiment 1, including all 375 objects and 125 scenes (i.e., only one exemplar of each scene). Following semantic criteria determined by consensus among the authors, each of the 125 scenes was paired with three semantically congruent objects as well as with three semantically incongruent objects. In this way, future studies may select and manipulate more than one congruent or one incongruent semantic relationship for each scene. See Fig. 3 for an example of the pairing between a scene and the corresponding six objects, and Table S3 on the online supplemental materials (https://osf.io/4pqsu/?view_only=9478429999754bd1afc3823c4876de18) for the description of all 125 septuplets. Each object was paired twice, once with a congruent scene and once with an incongruent scene.

#### Procedure

Participants were tested in small groups at the university laboratories. The experiment was built on the Qualtrics survey platform (Qualtrics, Provo, UT, USA). At the beginning of the experiment, after giving informed consent, participants provided sociodemographic information (i.e., age, gender, and native language).

Even though each scene has been paired with six objects, participants evaluated each object–scene pair at a time. On each trial, they saw two images side by side, one representing an object and another denoting a scene. They were asked to rate on a five-point Likert scale (1 = not related at all, 5 = highly related) how related those two items were or, in other words, how probable it was to find that object in that particular place. The experimenter provided oral examples (with items not included in the test) and participants responded at their own pace. The number of participants per pair varied between 23 and 26 (*M*_N_ = 24.3, *SD* = 1.0).

### Results and discussion

**Fig. 4:**
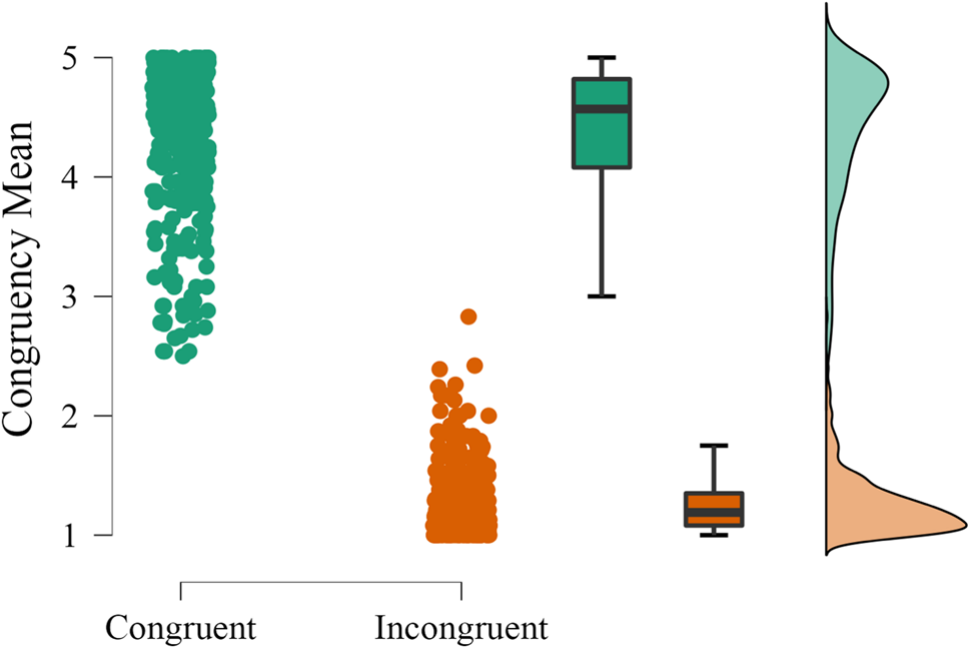
Distribution of the mean semantic congruency rating for the 375 congruent and 375 incongruent object–scene pairs. *Note*. The graph includes individual dots (corresponding to object–scene pairs), boxplots, and raincloud plots (Allen et al., 2019). Green: Congruent pairs. Orange: Incongruent pairs. For a detailed data overview, see Table S8 on the online supplemental materials (https://osf.io/4pqsu/?view_only=9478429999754bd1afc3823c4876de18)

Data preprocessing and analysis were performed on the congruency ratings obtained for each object–scene pair. Detailed information and descriptive statistics for each pair can be found in Table S5 of the supplemental materials (https://osf.io/4pqsu/?view_only=9478429999754bd1afc3823c4876de18). Overall, semantic congruency ratings (*M* = 2.81; *SD* = 1.62) varied along the whole five-point scale. An independent-sample *t*-test confirmed the expected difference, with higher rating scores for congruent than incongruent pairs (*t*(748) = 92.96, *p* < .001). Since these data represent a bimodal distribution, separate descriptive statistics for the 375 congruent and 375 incongruent relationships are depicted in Fig. 4.

Ratings of the congruent pairs had a larger range (2.50–5.00) than incongruent (1.00–2.83) pairs. Nonetheless, each range allows for a wide choice of semantic relationship values, according to the goals of future studies. On average, all incongruent pairs were judged below the midpoint of the scale. For the congruent set, 95% of the pairs were judged above the midpoint. For 18 out of the 375 congruent pairs, participants’ judgement was, on average, below the scale midpoint^1^. Besides, while the congruent rating distribution respected normality parameters (between ±2; Gravetter & Wallnau, 2014), incongruent relationships followed a more skewed-right and peaked distribution. This reveals that participants considered most of the incongruent pairs highly incongruent, as opposed to the congruent ones that showed more distributed ratings. The five-point Likert scale may have also contributed to this tendency (compared to a scale with more points), but we wanted to maintain the same number of scale points used for the other variables, and one that is common across studies (e.g., Adlington et al., 2009; Brodeur et al., 2010; Liu et al., 2011; Moreno-Martínez & Montoro, 2012; Paolieri & Marful., 2018; Rossion & Pourtois, 2004; Sirois et al., 2006; Snodgrass & Vanderwart, 1980).

We subsequently inspected potential correlations between the congruency ratings and the items’ parameters assessed in Experiment 1 (i.e., proportion of name agreement, familiarity, and visual complexity). We did not find any significant correlation for object pictures. As for scenes, there was only a significant correlation between the congruency ratings of the incongruent pairs and the scenes’ familiarity (*r*(125) = .21, *p* = .017), such that less familiar scenes were associated with more extreme incongruent ratings, even though this effect was weak.

## Experiment 3

### Method

#### Participants

A group of 26 young adults participated in this experiment (all female, *M*_age_ = 19.6 years, age range = 18–23 years), none of which participated in Experiments 1 and 2. They were all university students in Portugal. All participants provided oral informed consent, had European Portuguese as their native language, and had normal or corrected-to-normal vision. The experimental procedures were approved by the local ethics committee.

#### Stimuli

**Fig. 5:**
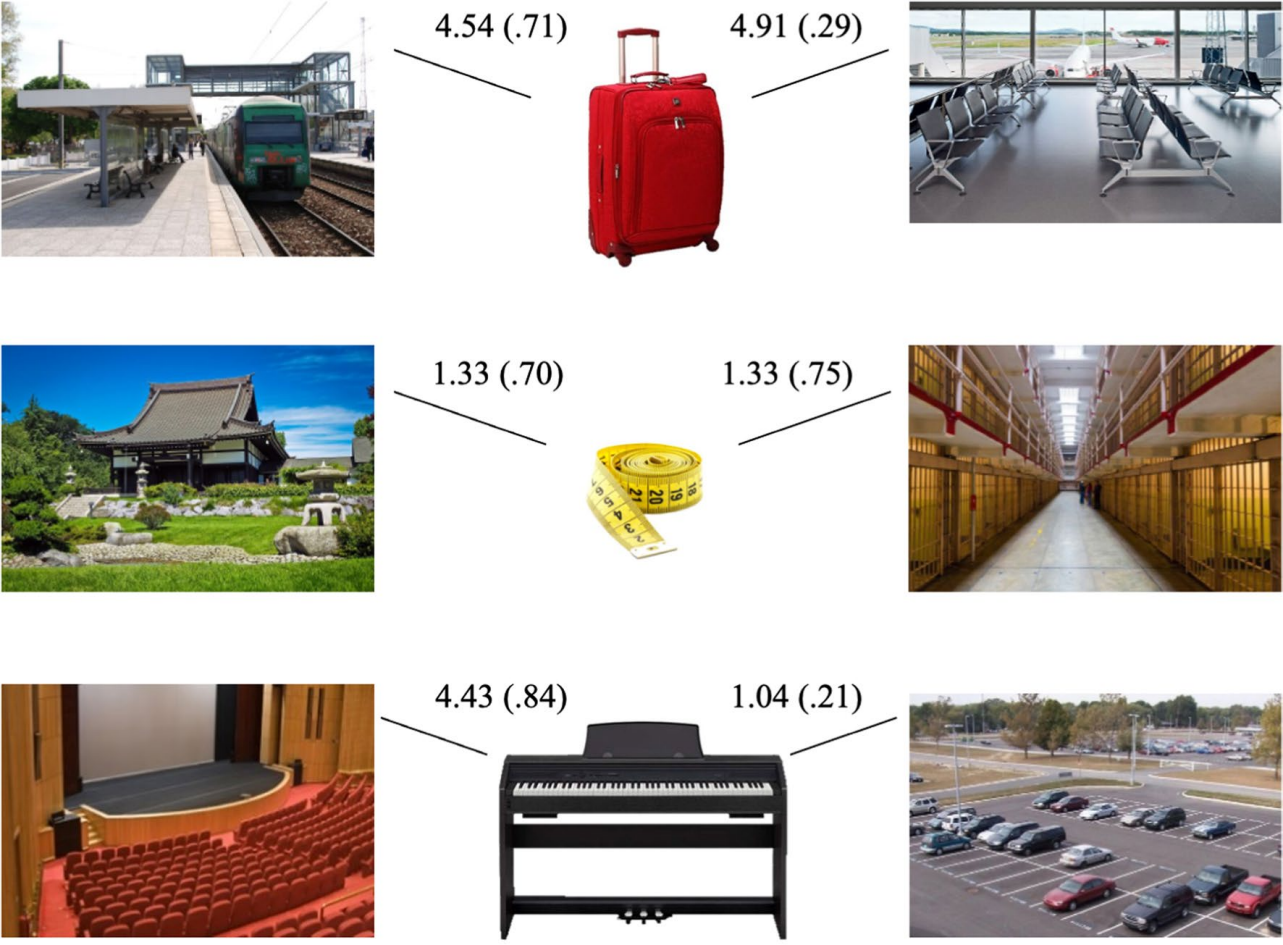
Illustration of the three types of triplets consisting of one object and two scenes. *Note.* Top row: Object (i.e., suitcase) paired with two congruent scenes (i.e., train station and airport). Middle row: Object (i.e., measuring tape) paired with two incongruent scenes (i.e., temple and prison). Bottom row: Object (i.e., digital piano) paired with a congruent scene (i.e., stage) and an incongruent scene (i.e., parking lot). Each value on the image represents the mean congruency rating (and standard deviation) for that particular object–scene pair

We used 360 pictures from Experiment 1, including 120 objects and 240 scenes (i.e., the two instances of each of the 120 scenes). Following semantic criteria consensual among the authors, each object was paired with two scenes, which always denoted two distinct places. See Fig. 5 for an example of the pairing between an object and the two corresponding scenes, and Table S4 of the supplemental materials (https://osf.io/4pqsu/?view_only=9478429999754bd1afc3823c4876de18) for the description of all 120 triplets. Overall, the pairing respected the following criteria: 30 objects were paired with two semantically congruent scenes (see top row of Fig. 5); 30 objects were paired with two semantically incongruent scenes (middle row of Fig. 5); 60 objects were paired with a semantically congruent and a semantically incongruent scene (bottom row of Fig. 5). We decided to double the number of trials in the latter condition, to allow for future studies to separate these trials into two subsets. For example, one subset in which objects are paired with a target scene that is congruent and with a distractor scene that is incongruent, while in another subset, objects are paired with an incongruent target scene and a congruent distractor scene. As such, this pairing allows for full factorial designs with congruent and incongruent targets and congruent and incongruent distractors.

In 40% of the pairings, the two instances of the same scene belonged to the same semantic congruency condition (i.e., either both congruent or both incongruent pairs). For example, one exemplar of “pharmacy” was paired with a thermometer and the other exemplar with pills, thus forming two congruent pairs. In the remaining 60% of the trials, the two exemplars were paired with objects creating a congruent and an incongruent relationship. For instance, one exemplar of “casino” was paired with a dice and the other exemplar with an anchor.

Even though the pairing in this experiment was new (relative to the one made in Experiment 2), 92 pairs had already been evaluated in the previous experiment. Thus, in this experiment, participants evaluated only 148 of the 240 relationships at stake.

#### Procedure

Participants were tested in small groups at the university laboratories. The experiment was built in E-Prime software, and participants responded using the keyboard of the computer. At the beginning of the experiment, after giving informed consent, participants provided sociodemographic information (i.e., age, gender, and native language).

The procedure was similar to the one used in Experiment 2, with participants evaluating each object–scene pair at a time. The number of participants that responded to each pair varied between 23 and 24 (*M*_N_ = 23.9, *SD* = 0.3).

### Results and discussion

**Fig. 6:**
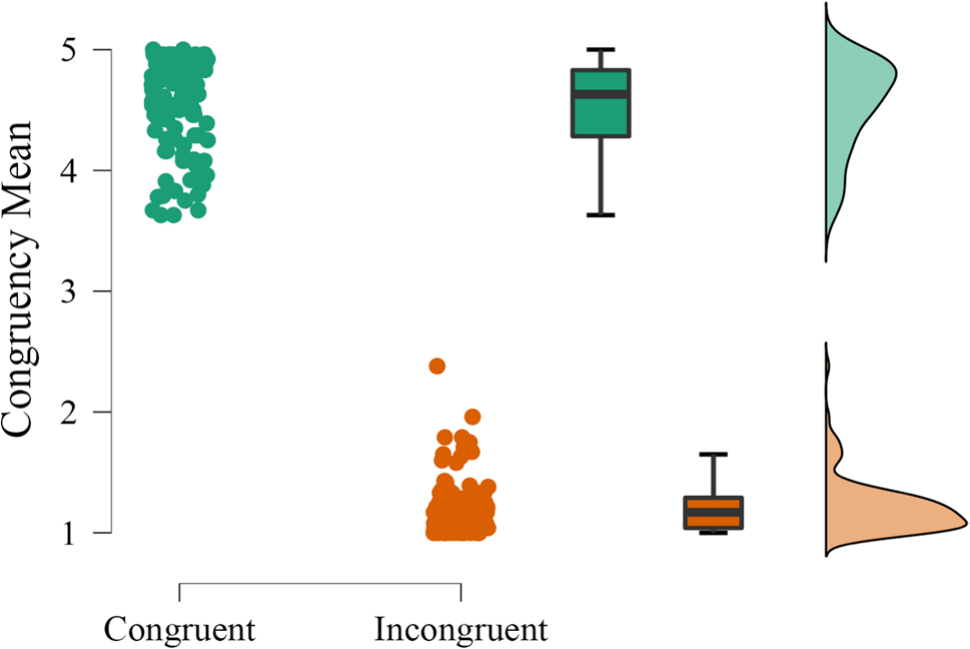
Distribution of the mean semantic congruency rating for the 120 congruent and the 120 incongruent object–scene pairs. *Note*. The graph includes individual dots (corresponding to object–scene pairs), boxplots, and raincloud plots (Allen et al., 2019). Green: Congruent pairs. Orange: Incongruent pairs. For a detailed data overview, see Table S9 on the online supplemental materials (https://osf.io/4pqsu/?view_only=9478429999754bd1afc3823c4876de18)

Data preprocessing was conducted on the congruency ratings for each 148 object–scene pairs tested. As mentioned above, these responses were then analyzed together with the 92 pairs previously validated in Experiment 2. Detailed information and descriptive statistics for each pair can be found in Table S5 of the online supplemental materials (https://osf.io/4pqsu/?view_only=9478429999754bd1afc3823c4876de18). Overall, congruency ratings for the 240 semantic relationships (*M* = 2.87; *SD* = 1.67) varied along the entire five-point scale. An independent-samples *t*-test confirmed that congruent pairs were rated significantly higher in congruency than incongruent pairs (*t*(238) = 83.77, *p* < .001). Separate descriptive statistics for the 120 congruent and 120 incongruent pairs are depicted in Fig. 6.

In contrast to Experiment 2, congruent and incongruent judgments presented a similar range (3.63–5.00 and 1.00–2.38, respectively). On average, all congruent pairs were judged above the midpoint of the scale, and the opposite was seen for incongruent pairs. Regarding the congruent and incongruent distributions, the pattern was similar to Experiment 2, i.e., congruent relationships were normally distributed but incongruent relationships revealed more extreme values of incongruency.

As in Experiment 2, we looked for potential correlations between congruency ratings of congruent and incongruent pairs and the ratings of items’ parameters assessed during Experiment 1. We did not find any significant correlation for object pictures. There was a weak but significant correlation between the mean congruency rating of incongruent pairs and the scenes’ name agreement (*r*(120) = −.26, *p* = .004), suggesting that scenes with higher name agreement were associated with more extreme values of incongruency.

## Conclusions

The pictorial stimulus set presented here gathered normative ratings on the same variables for object and scene pictures, including measures of name agreement, familiarity, and visual complexity, hence offering comparable measures for both types of pictures. Moreover, it provides normative data for semantic congruency ratings of object–scene picture pairs that are presented separately. Experiment 1 presents norms of name agreement, familiarity, and visual complexity for 620 individual objects and scenes. Across Experiments 2 and 3, participants rated the semantic relationship of 898 object–scene pairs, including 433 congruent and 465 incongruent pairs. To our knowledge, this is the first database validating semantic relationships between pairs of object and scene pictures that are presented separately. In this way, the database offers flexibility in the use of pictures that can be employed in isolation or combined. Databases of objects embedded within background scenes constitute more ecologically valid stimuli for some research (e.g., Bar, 2004; Bates et al., 2003; Duñabeitia et al., 2018; Hebart et al., 2019; Krautz & Keuleers, 2022; Szekely et al., 2004; Võ, 2021). Yet, our database allows one disentangle the processing of the object and its context (the scene), being more suited to investigations in episodic memory and psycholinguistics. In addition, in contrast to previous databases, we ensured that none of the images portray human faces, actions, or letters/words, and that the objects selected do not appear in the scene pictures, preventing both the interference from additional cognitive processes and item repetition.

Each scene was paired with six objects (three congruent and three incongruent), forming the picture septuplets, and we provide the semantic relationship judgment for each of these pairs. Similarly, each object was paired with two scenes (in congruent and incongruent relationships), forming the picture triplets, and we provide the respective norms. According to Bar (2004), an object can cognitively activate the context in which this object might appear, and similarly, a scene may activate the objects that usually appear in that context. With this in mind, we believe it is useful for future studies that this database provides not only scenes associated with congruent and incongruent objects (septuplets of Experiment 2), but also objects paired with congruent and incongruent scenes (triplets of Experiment 3). The organization of the material with multiple pairs per stimulus allows future research to select various semantic relationships for the same item (object or scene). In addition, by having two different exemplars for most scenes (e.g., two kitchens), it is possible to use this material to unravel the processing of a target scene versus a related lure (e.g., in a two-alternative forced choice paradigm as in Konkle et al., 2010).

A drawback of our stimulus set is that we did not control for low-level features of the images (e.g., contrast, luminance). It would be beneficial for future research to assess these properties, and in this way complement the subjective judgments made by the participants with these more objective low-level properties. In addition, sample sizes of the experiments were determined based on previous studies (e.g., Denkinger & Koutstaal, 2014; Johnston et al., 2010; Öhlschläger & Võ, 2017) and according to the resources available. Yet, a prior power analysis to estimate these sample sizes would have been more adequate to ensure that the experiments are sufficiently powered to detect potential differences. Despite this limitation, to allow comparison between experiments, we kept the number of participants that judged each image relatively constant across experiments. Specifically, in Experiment 1, an average of 22.1 subjects judged each object picture while an average of 27.2 subjects judged each scene image. In Experiment 2, each object–scene pair was judged by an average of 24.3 participants. In Experiment 3, the number of participants that responded to each pair was, on average, 23.9. Another limitation to consider is that familiarity and name agreement have a potentially limited generalization as they vary culturally (e.g., Umla-Runge et al., 2012) and therefore generalization of these data to other cultures or groups should be taken with caution. In the three experiments, participants were native speakers of European Portuguese. As such, the validation and use of these materials in other cultures is an important endeavor.

The criterion adopted for the images’ size of objects was based on the proportional size of objects in real life. In some studies, such as in language comprehension or in reaction-time experiments, this may constitute a caveat, with earlier studies showing that an object’s size may modulate the visual integration of other features (e.g., Chen et al., 2020; Plewan et al., 2012). However, we opted to follow the rationale of the “familiar size” rule of the object’s organization in the real-world (Biederman et al., 1982), which highlights that objects are drawn, imagined, and viewed at their size proportion in the real world (Konkle & Oliva, 2011), which may constitute an advantage for some research fields (such as in episodic memory studies).

Nonetheless, the *ObScene Database* constitutes a highly flexible, diverse, and controlled stimulus set, and a suitable tool to employ in future human cognition studies, either using the objects and the scenes independently or the picture septuplets and triplets that we built. Besides its relevance for object/scene perception and recognition studies, this database can be of great value for research in psycholinguistics using word/sentence–picture tasks or for memory studies investigating, for example, item memory, context memory, and the relationship between the two mnemonic processes.

## Data Availability

The materials for all experiments and their corresponding detailed data are available at https://osf.io/4pqsu/?view_only=9478429999754bd1afc3823c4876de18. This study was not preregistered.

## Footnotes

1 In Tables S3 and S4 of the supplemental materials (https://osf.io/4pqsu/?view_only=9478429999754bd1afc3823c4876de18), the 18 congruent object–scene pairs with a mean congruency rating below the midpoint of the scale are coded with the following Relationship Numbers: 39; 51; 286; 296; 300; 301; 303; 315; 317; 338; 359; 539; 555; 572; 586; 608; 620; 623.
